# On Painful Distension of the Vagina after the Birth of a Child

**Published:** 1853-08

**Authors:** 


### OBSTETRICS.

*On Painful Distension of the Vagina after the Birth of a Child.* By Dr. LEOPOLD.—Dr. Leopold states that he has several times met with examples (never in primiparæ) of excessive suffering, coming on from half an hour to an hour after the passage of the child, and referred to the vagina. It is of the most agonizing character, described by the women as worse than when the child is passing, and causing them to twist and toss about in agony. It arises from distension of the vagina, either by accumulated coagula, or a very large placenta. In the first case, owing to the quantity of blood lost, there are always the early symptoms of uterine hæmorrhage; and the women, usually having suffered already from hæmorrhage in former labors, are dreadfully frightened at their danger. The softish but not distended uterus is felt pushed up into the umbilical region. The hand should be at once introduced into the vagina, and after the coagula are completely removed, it should be retained there for at least half an hour, keeping two fingers on the watch within the relaxed os uteri, and irritating this if required.

In the other case there is always a large placenta, which will not yield to moderate traction, partly because its size prevents it from easily traversing the vaginal passage, partly from a spasmodic action of the *constrictor cunni*, and partly because the membranes still remain in connection with the uterus to some extent. Notwithstanding the pain it will cause, the entire hand must be passed into the vagina, so as to embrace the whole placenta and bring it down.—*Neue Zeitschrift für Geburtskinde*, from *Brit. and For. Med.-Chi. Rev.*

